# Chinese Herbal Formula Huayu-Qiangshen-Tongbi Decoction Attenuates Rheumatoid Arthritis through Upregulating miR-125b to Suppress NF-*κ*B-Induced Inflammation by Targeting CK2

**DOI:** 10.1155/2022/2836128

**Published:** 2022-07-04

**Authors:** Xiu-min Chen, Kai-xin Gao, Xiao-Dong Wu, Huang-sheng Liang, Ze-hao Liu, Mao-jie Wang, Li-yan Mei, Qing-chun Huang, Run-yue Huang

**Affiliations:** ^1^State Key Laboratory of Dampness Syndrome of Chinese Medicine, The Second Affiliated Hospital of Guangzhou University of Chinese Medicine, Guangzhou, China; ^2^Department of Rheumatology, The Second Affiliated Hospital of Guangzhou University of Chinese Medicine, Guangzhou, China; ^3^Guangdong Provincial Key Laboratory of Chinese Medicine for Prevention and Treatment of Refractory Chronic Diseases, Guangzhou, China; ^4^Guangdong-Hong Kong-Macau Joint Lab on Chinese Medicine and Immune Disease Research, Guangzhou, China; ^5^Second Clinical Medical College, Guangzhou University of Chinese Medicine, Guangzhou, Guangdong, China

## Abstract

The Huayu-Qiangshen-Tongbi (HQT) decoction, a Chinese medical formula, has been identified to show a potent therapeutic effect on rheumatoid arthritis (RA). However, the specific molecular mechanism of HQT in RA has not been well studied. In the present study, LPS-treated human rheumatoid fibroblast-like synoviocyte (FLS) MH7A cells and collagen-induced arthritis (CIA) mice were utilized as *in vitro* and *in vivo* models. Our results demonstrated that HQT could efficiently inhibit RA-induced inflammation by reducing the production of cytokines including tumor necrosis factor alpha (TNF-*α*), interleukin-1 beta (IL-1*β*), and interleukin-6 (IL-6). Moreover, HQT significantly upregulated the expression of miR-125b. Besides, analysis of bioinformatics suggested casein kinase 2 (CK2) was a potential target of miR-125b. Luciferase reporter assay was performed and revealed that miR-125b suppressed CK2 expression in MH7A cells. Furthermore, miR-125b inhibited LPS-induced NF-kappa-B (NF-*κ*B) activation, which is a downstream target of CK2. In addition, the NF-*κ*B inhibitor ammonium pyrrolidinedithiocarbamate (PDTC) and NF-kappa-B inhibitor alpha (IkB-*α*) enhanced the inhibitory effect of miR-125b on the expression of TNF-*α*, IL-1*β*, and IL-6. Taken together, our study revealed that HQT could attenuate RA through upregulating miR-125b to suppress NF-*κ*B-induced inflammation by targeting CK2. The findings of this study should facilitate investigating the mechanism of HQT on RA and discovering novel therapeutic targets for RA.

## 1. Introduction

Rheumatoid arthritis (RA) has been identified as a chronic inflammatory disease characterized by painful, swollen joints, which leads to disability and deteriorates the physical function and quality of life [1–3]. About 1 out of 100 people suffer RA during their lifetime globally [4]. In addition, RA can increase the risk of other severe diseases, such as cardiovascular diseases [5]. To date, disease-modifying antirheumatic drugs (DMARDs) are required for the treatment for RA [6]. However, unwanted side effects including myelosuppression, liver injury, and kidney injury greatly limit the efficiency of DMARDs [7]. Therefore, it is urgent to discover novel drugs for RA treatment.

The Huayu-Qiangshen-Tongbi (HQT) decoction, a Chinese medical formula developed by the Second Affiliated Hospital of Guangzhou University of Chinese Medicine, is composed of a total of 10 herbs and has been demonstrated to exert a potent therapeutic effect on RA [8, 9]. Moreover, our previous study has revealed that the combined therapy of HQT and methotrexate (MTX) exerts definite clinical effects on RA with less adverse effects in 45 patients with RA [9, 10]. MTX is an essential medicine that is applicated as a first-line treatment for RA [11]. Although HQT is widely used for the treatment of RA in China, the molecular mechanism on how HQT attenuates RA remains unclear, which limits the global utilization of HQT for RA treatment. Hence, the mechanism under which HQT ameliorates RA needs to be investigated.

MicroRNAs (miRNAs) are a class of noncoding RNAs with approximately 22 nucleotides in length, which regulate cellular and physiological processes in various diseases by targeting mRNAs at the posttranscriptional level [12, 13]. In the last decades, numerous miRNAs have been reported to play crucial roles in the progression of RA. For example, miR-126 inhibits TNF-*α* production through targeting of the interleukin-23 receptor (IL-23R) in the fibroblast-like synoviocytes (FLSs) of RA mice [14]. Moreover, miR-506 has been identified to suppress proliferation and induces apoptosis of FLS in RA by targeting Toll-like receptor 4 (TLR4) [15]. Besides, miR-34a-3p prohibits cell proliferation and reduces the production of proinflammatory cytokines by targeting murine double minute 4 (MDM4) in FLS collected from RA patients [15]. Furthermore, miR-138 has been demonstrated to induce the production of inflammatory cytokines through activating NF-*κ*B and granulin precursor (GRN) via directly targeting histone deacetylase 4 (HDAC4) [16]. In addition, our recent study has indicated that HQT may attenuate RA through modulating miR-19b via long noncoding RNA (lncRNA) uc.477 [17].

A previous study has revealed that miR-125b is significantly downregulated in RA patients [18]. miR-125b has been reported to regulate inflammation in various human diseases. For instance, the dysregulation of miR-125b may lead to the impaired B-cell responses in Down Syndrome (DS) [19]. Moreover, it has been identified that miR-125b modifies monocyte adaptation to inflammation via mitochondrial metabolism and dynamics [20]. Furthermore, miR-125b could activate the NF-*κ*B pathway through targeting the tumor necrosis factor alpha-induced protein 3 (TNFAIP3) [21]. However, the role of miR-125b in RA remains unknown.

Protein kinase CK2 is a critical regulator of immunity. For instance, CK2 is an emerging regulator of T-cell-driven autoimmune disorders, such as multiple sclerosis (MS) [22]. Besides, myeloid cell CK2 induces protective innate immune responses against bacterial infection [23]. CK2 also plays a crucial role in inflammatory disorders of cancers. In breast cancer, CK2 enhances IL-6 production to promote tumor progression [24]. In addition, CK2 is a positive regulator of NF-*κ*B [25, 26], which is a positive regulator of RA through inducing inflammatory disorders. However, the effect of CK2 on RA is not clear.

It was hypothesized that HQT may attenuate RA through inhibiting the NF-*κ*B pathway via the miR-125b/CK2 axis. Therefore, the primary aim of this study was to investigate the mechanism on how HQT ameliorates RA progression.

## 2. Materials and Methods

### 2.1. Collagen-Induced Arthritis (CIA) Rat Model

All animal procedures were performed according to National Institutes of Health guidelines and approved by the Ethics Committee of the Second Affiliated Hospital of Guangzhou University of Chinese Medicine. Wistar rats (male, approximately 6-8 weeks) were purchased from the Beijing Vital River Laboratory Animal Technology Co., Ltd., and kept under specific pathogen-free conditions. The collagen-induced RA mouse model was established as previously described with minor modification [27]. In brief, bovine type II collagen (Chondrex, Redmond, Washington, USA) was fixed with incomplete Freund's adjuvant (Chondrex) at ratio 1 : 1 and homogenized for 30 minutes by using a homogenizer. At the beginning of the experiments (day 0), rats were immunized with a 0.1 mL emulsion containing 100 *μ*g of collagen at the tail base, and then, immunity was strengthened once on the eighth day. Day 1 was recorded as the first modeling day, and the drug was administered for 30 consecutive days. Rats were divided into six experimental groups (*n* = 8): control group (saline), model group (CIA), MTX group (MTX, 1 mg·kg^−1^), HQT-L (CIA rats received 9.4 g/kg HQT, low dose), HQT-M (CIA rats received 18.8 g/kg HQT, medium dose), and HQT-H (CIA rats received 37.6 g/kg HQT, high dose). After 30 days, rats were euthanatized, and the joint diameters were measured with a pocket thickness gauge to evaluate the ankle joint swelling. Meanwhile, the blood was collected from rats and used for the subsequent qRT-PCR and ELISA analyses.

### 2.2. Cell Culture

Human rheumatoid fibroblast-like synoviocyte (FLS) MH7A cells were obtained from the American Type Culture Collection (ATCC, Manassas, VA, USA) and cultured with DMEM supplemented with 10% FBS (Gibco, Carlsbad, CA, USA), 100 U/mL penicillin, and 100 *μ*g/mL streptomycin (Gibco) at 37°C with 5% CO_2_. When needed, additional HQT was added and incubated.

### 2.3. LPS-Induced Inflammation Cell Model

LPS (L2880, Sigma, St. Louis, MO, USA) was dissolved in PBS to make a stock solution (5 mg/mL) by sonication for 2 min, after which aliquots were obtained and stored at −80°C until use. LPS-induced inflammation cell model was constructed as previously described [28]. Briefly, MH7A cells were pretreated with 0.1 mg/mL HQT in a serum-free medium for 2 h and then persistently incubated for another 24 h with or without subsequent exposure to 1 *μ*g/mL LPS. For the inflammatory cytokine detection and miR-125b expression, qRT-PCR analysis and ELISA assay were applied. When needed, NF-*κ*B inhibitor PDTC (S3633, Selleck, Shanghai, China) was added.

### 2.4. Cell Transfection

miR-125b mimics, miR-125b inhibitor, and respective negative controls (miR-NC and inhibitor NC) (GenePharma, Shanghai, China) were transfected into MH7A cells via the Lipofectamine 2000 kit (Invitrogen, Carlsbad, CA, USA) according to the manufacturer's instructions. For the IkB-*α* overexpression, the cDNA sequence of IkB-*α* was obtained via PCR amplification and then cloned into the pcDNA3.1 expression vector. After transfection for 48 h, cells were collected and used for the subsequent experiments. The sequence used in this study is as follows: miR-125b mimic forward: 5′-UCCCUGAGACCCUAACUUGUGA-3′, reverse: 5′-ACAAGUUAGGGUCUCAGGGAUU-3′; miR-NC forward: 5′-UUCUCCGAACGUGUCACGUTT-3′, reverse: 5′-ACGUGACACGUUCGGAGAATT-3′; miR-125b inhibitor: 5′-UCACAAGUUAGGGUCUCAGGGA-3′; and inhibitor NC: 5′-CAGUACUUUUGUGUAGUACAA-3′.

### 2.5. CCK-8 Assay

Cell viability was evaluated by using the Cell Counting Kit-8 (Dojindo, Beijing, China) according to the manufacturer's instructions as previously described [29]. In brief, 1 × 10^4^ cells were seeded into 96-well plates and treated with different concentrations of HQT such as 0, 0.1, 1, 10, and 100 mg/mL for 24 h; the supernatant was removed; and 10 *μ*L of CCK-8 solution was added to each well, and the absorbance was detected using a microplate reader (Thermo Fisher Scientific, Cleveland, OH, USA) at 450 nm.

### 2.6. Luciferase Reporter Assay

The wild type (WT) and mutant (MUT) of CK2 containing the putative binding site with miR-125b were amplified via PCR and cloned into the pmirGLO luciferase reporter vector (Promega Corporation, Madison, WI, USA). Then, the luciferase reporter plasmids were cotransfected with miR-125b mimics, miR-NC, miR-125b inhibitor, or inhibitor NC into MH7A cells by using a Lipofectamine 2000 kit. Following 48 h transfection, relative luciferase activity was determined using the dual-luciferase reporter assay system with Renilla luciferase activity as the internal control.

### 2.7. qRT-PCR Analysis

Total RNA of blood or cultured cells was extracted by using the TRIzol reagent (Invitrogen). Total RNA was reversely transcribed to cDNA via the PrimeScript II 1st Strand cDNA Synthesis Kit (Takara Bio, Inc., Dalian, Liaoning, China), and qRT-PCR analysis was performed using the SYBR Premix Ex Taq II (Takara Bio, Inc.) based on the 7500 Fast Real-Time PCR System (Applied Biosystems, Foster City, CA, USA). The relative fold expression change of target genes was analyzed by using the 2^−*ΔΔ*Ct^ method [30], with U6 as the internal reference. The primers used in this study are as follows: miR-125b forward: 5′-TCCCTGAGACCCTAACTTGTGA-3′, reverse: universal primer (miScript SYBR-Green PCR kit); pre-miR-125b forward: 5′-TGCGCTCCTCTCAGTCCC, reverse: 5′-TGGGAGCTGCGAGTCGTG-3′; pri-miR-125b forward: 5′-CGAACAGAAATTGCCTGTCA-3′, reverse: 5′-ACCAAATTTCCAGGATGCAA-3′; and U6 forward: 5′-CTCGCTTCGGCAGCACA-3′, 5′-reverse: AACGCTTCACGAATTTGCGT-3′.

### 2.8. Western Blot

Total protein of cultured cells was extracted using the RIPA lysis buffer containing protease inhibitor PMSF. Subsequently, approximately equal amounts of protein were separated via 10% SDS-PAGE and transferred into PCDF membranes. After blocking with 5% nonfat milk for 5 min, the membranes were then incubated with primary antibody anti-p-p65 (1 : 500, bs-5660R, Bioss, Beijing, China), anti-p-IkB-*α* (1 : 500, bs-3191R, Bioss), and anti-GAPDH (1 : 10000, KC-5G5, Aksomics, Shanghai, China) at 4°C overnight. After incubation with an appropriate HRP-conjugated secondary antibody for 1 h at room temperature, membranes were extensively washed. Finally, the bonds were visualized by using the Amersham ECL kits (GE Healthcare, London, UK), and the optical density of the protein bands was quantified under ImageJ software.

### 2.9. Enzyme-Linked Immunosorbent Assay (ELISA) Assay

Cytokine concentration including TNF-*α*, IL-1*β*, and IL-6, as well as human CK2 concentration in serum or cellular supernatant, was measured using the ELISA kit, of which, the rat TNF-*α* ELISA kit (DG20065D), mouse IL-1*β* ELISA kit (DG94484Q), mouse IL-6 ELISA kit (DG94490Q), human casein kinase 2 (CK2) ELISA kit (DG95793Q), human TNF-*α* ELISA kit (DG10053H), human IL-1*β* ELISA kit (DG10307H), and human IL-6 ELISA kit (DG10306H) were all purchased from the Beijing Winter Song Boye Biotechnology Co. Ltd. (China).

### 2.10. Statistical Analysis

Data were analyzed via SPSS19.0 statistical software and presented as the mean ± SD method. All experiments were repeated as least three times. Difference was determined by Student *t* test (two groups) or two-way ANOVA (multiple groups). *p* value < 0.05 was considered as the significant threshold.

## 3. Results

### 3.1. HQT Attenuates CIA-Induced Inflammation and Upregulates miR-125b in Rats

**Figure 1:**
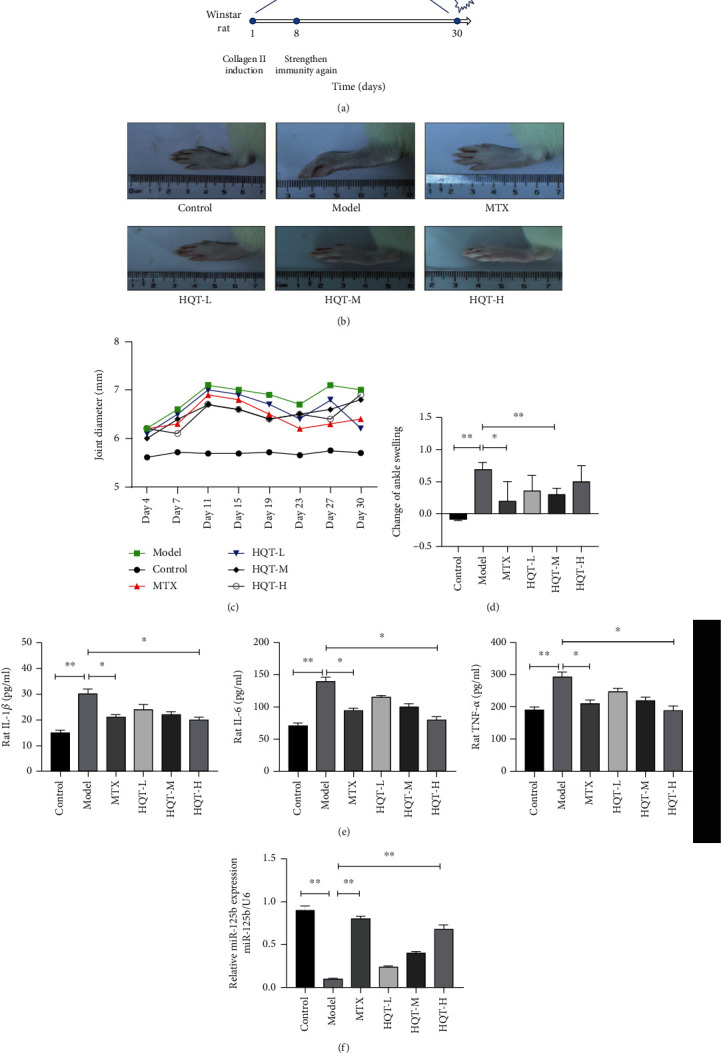
HQT attenuated RA inflammation by modulating miR-125b. Mice were divided into six groups: control group (saline), model group (CIA induction), MTX group (MTX, 1 mg·kg^−1^), HQT-L (CIA mice that received low dose HQT of 9.4 g/kg, low dose), HQT-M (CIA mice that received medium dose HQT of 18.8 g/kg, medium dose), and HQT-H (CIA mice that received high dose HQT of 37.6 g/kg, high dose). (a) Schematic diagram of the study. (b) Representative pictures of the mouse hind paw on day 30. (c) Joint swelling was assessed by measuring the ankle joint diameter with a pocket thickness gauge (*n* = 8). (d) Change of ankle swelling was analyzed. (e) The cytokine protein levels including TNF-*α*, IL-1*β*, and IL-6 in the serum of mice from different groups were measured by using an ELISA assay (*n* = 8). (f) The mRNA expression of miR-125b in the serum of mice from different groups was evaluated by qRT-PCR (*n* = 8). ^∗^*p* < 0.05, ^∗∗^*p* < 0.01, and ^∗∗∗^*p* < 0.001.

Firstly, we explored the roles of HQT in collagen-induced arthritis (CIA) with MTX as the positive control, and the schematic diagram is shown in Figure 1(a). As shown in Figure 1(b), the joint edema of CIA rats (model group) was obviously exacerbated compared with that of the control group, and MTX treatment markedly relieved joint edema compared with the model group (Figure 1(b)). Besides, different doses of HQT including low-, medium-, and high-dose HQT could significantly ameliorate joint edema compared with the model group (Figure 1(b)). Furthermore, the ankle joint swelling was evaluated by using the ankle joint diameter, and the results indicated that the ankle joint diameter of CIA rats was significantly increased compared with that of the control group (*p* < 0.05), and MTX treatment obviously decreased the ankle joint diameter compared with the model mice (*p* < 0.05) (Figure 1(c)). Similarly, HQT also relieved the ankle joint swelling compared with the model mice (*p* < 0.05), while low-dose HQT exhibited the thinnest ankle joint diameter on day 30 (Figure 1(c)). The arthritic change of ankle swelling between different groups was analyzed, and the results demonstrated that MTX and medium-dose HQT could efficiently attenuate CIA-induced ankle joint swelling (*p* < 0.05) (Figure 1(d)).

Next, the levels of serum inflammation-related cytokines in rats were measured via ELISA assay, and the results revealed that the levels of serum TNF-*α*, IL-1*β*, and IL-6 were significantly increased in the model group compared with the control group (*p* < 0.01), while MTX and HQT treatments dramatically reduced the levels of serum TNF-*α*, IL-1*β*, and IL-6 compared with the model group (*p* < 0.05) (Figure 1(e)).

Interestingly, the level of serum miR-125b in the model group was significantly downregulated compared with that in the control group (*p* < 0.01), whereas MTX treatment markedly increased the serum miR-125b level compared with the model group (*p* < 0.01) (Figure 1(f)). Besides, HQT also increased the level of serum miR-125b compared with the model group in a dose-dependent manner (*p* < 0.05) (Figure 1(f)). These results suggested that HQT might attenuate inflammation and upregulate miR-125b in RA.

### 3.2. CK2 Is a Target of miR-125b in MH7A Cells

**Figure 2:**
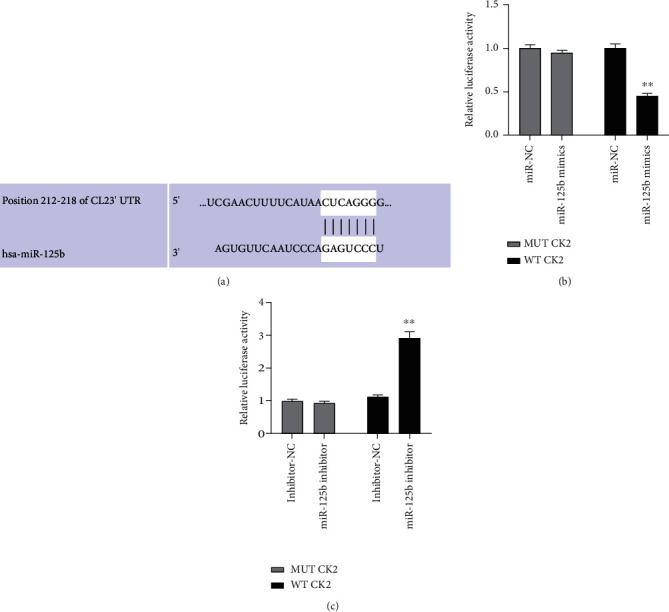
CK2 is a target of miR-125b in MH7A cells. (a) The putative binding site between miR-125b and CK2 was predicted by TargetScan. (b, c) MH7A cells were transfected with miR-125b mimics or miR-NC (b) and miR-125b inhibitor or inhibitor NC (c); then, the relative luciferase activity of WT or MUT CK2 was evaluated by using a dual luciferase reporter system. ^∗^*p* < 0.05, ^∗∗^*p* < 0.01, and ^∗∗∗^*p* < 0.001.

To further explore the molecular mechanism of HQT in RA, the potential targets of miR-125b in human was predicted using TargetScan (http://www.targetscan.org/vert_71/). The result showed that there was a putative binding site between miR-125b and CK2 mRNA (Figure 2(a)), suggesting that CK2 might be a direct target of miR-125b.

To determine the correlation of miR-125b and CK2, miR-125b mimics or miR-NC was cotransfected with the luciferase reporter plasmid-contained WT 3′UTR of CK2 mRNA (WT CK2) or mutated 3′UTR of CK2 mRNA against the predictive binding site with miR-125b (MUT CK2) into MH7A cells to perform the luciferase reporter assay. Results indicated that the relative luciferase activity of cells transfected with the WT CK2 vector was decreased via cotransfection with miR-125b mimics compared with cells cotransfected with WT CK2 vector and miR-NC (*p* < 0.01) (Figure 2(b)). By contrast, the relative luciferase activity of cells transfected with the WT CK2 vector was increased via cotransfection with miR-125b inhibitors compared with that of cells cotransfected with the WT CK2 vector and inhibitor-NC (*p* < 0.01) (Figure 2(c)). However, the relative luciferase activity of cells transfected with MUT CK2 vector was not affected via cotransfection with miR-125b mimics or inhibitors (Figures 2(b) and 2(c)). These results suggested that CK2 is a direct target of miR-125b in MH7A cells.

### 3.3. miR-125b Suppresses NF-*κ*B Activation through CK2 in LPS-Treated MH7A Cells

**Figure 3:**
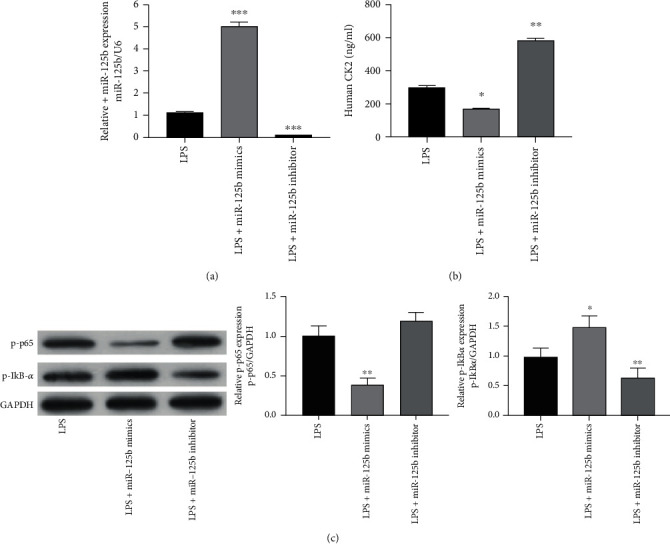
miR-125b suppresses NF-*κ*B activation through CK2 in LPS-treated MH7A cells. MH7A cells were transfected with miR-125b mimics or miR-125b inhibitor and then treated with 1 *μ*g/mL LPS for 24 h. (a) The mRNA expression of miR-125b was evaluated via qRT-PCR. (b) Human CK2 concentration in the cultured supernatant was measured by using an ELISA assay. (c) The protein expression of NF-*κ*B pathway members including p-p65 and p-IkB-*α* was evaluated via western blot. *N* = 3, ^∗^*p* < 0.05, ^∗∗^*p* < 0.01, and ^∗∗∗^*p* < 0.001.

To mimic RA *in vitro*, MH7A cells were treated with LPS. The qRT-PCR assay indicated that LPS significantly reduced the miR-125b expression in MH7A cells, while miR-125b mimics increased the level of miR-125b in LPS-treated MH7A cells (*p* < 0.001) (*p* < 0.001) (Figure 3(a)). Moreover, miR-125b mimics markedly decreased the protein level of CK2 in LPS-treated MH7A cells (*p* < 0.05), whereas the miR-125b inhibitor increased the CK2 protein expression in LPS-treated MH7A cells (*p* < 0.01) (Figure 3(b)).

NF-*κ*B is the downstream target of CK2. To identify whether the NF-*κ*B activation was regulated by miR-125, Western blot was performed. Results of Western blot showed that miR-125b mimics significantly decreased the level of phospho-NF-*κ*B p65 (p-p65) and increased the phospho-IkB-*α* (p-IkB-*α*) level in MH7A cells treated with LPS (*p* < 0.01), while the miR-125b inhibitor decreased the level of p-IkB-*α* (*p* < 0.05) and slightly increased the p-p65 level in LPS-treated MH7A cells (Figure 3(c)). These data suggested that miR-125b inhibited NF-*κ*B activation through CK2 in LPS-treated MH7A cells.

### 3.4. miR-125b Attenuates LPS-Induced Inflammation by Suppressing NF-*κ*B in MH7A Cells

**Figure 4:**
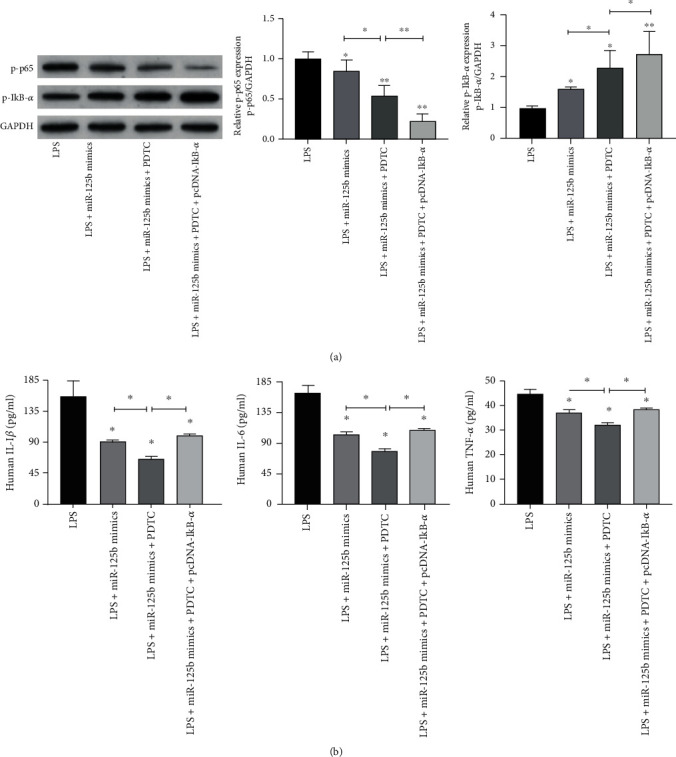
miR-125b suppresses NF-*κ*B activation through CK2 in LPS-treated MH7A cells. MH7A cells were transfected with miR-125b mimics or cotransfected with miR-125b and pcDNA-IkB-*α* and then treated with LPS or PDTC. (a) The protein level of p-p65 and p-IkB-*α* was evaluated via western blot. (b) The cytokine protein levels including TNF-*α*, IL-1*β*, and IL-6 in the cellular supernatant were measured by using ELISA assay. *N* = 3, ^∗^*p* < 0.05, ^∗∗^*p* < 0.01, and ^∗∗∗^*p* < 0.001.

To determine whether miR-125b attenuated LPS-induced inflammation via NF-*κ*B, the specific NF-*κ*B inhibitor PDTC was applied and IkB-*α* was overexpressed via transfection of pcDNA-IkB-*α*. Results of the Western blot showed that miR-125b mimics significantly reduced the level of p-p65 and increased the p-IkB-*α* level in MH7A cells treated with LPS (*p* < 0.05), while PDTC treatment and overexpression of IkB-*α* further enhanced the inhibitory effect of miR-125b mimics on the levels of p-p65 and p-IkB-*α* (*p* < 0.05) (Figure 4(a)). Besides, miR-125b mimics significantly decreased the levels of TNF-*α*, IL-1*β*, and IL-6 in LPS-treated MH7A cells (*p* < 0.05), and PDTC treatment and overexpression of IkB-*α* further improved the inhibitory effect of miR-125b mimics on the production of TNF-*α*, IL-1*β*, and IL-6 (*p* < 0.05) (Figure 4(b)). These results indicated that miR-125b suppressed LPS-induced inflammation by inhibiting NF-*κ*B in MH7A cells.

### 3.5. HQT Ameliorates LPS-Induced Inflammation through Upregulating miR-125b to Suppress NF-*κ*B-Induced Inflammation by Targeting CK2

**Figure 5:**
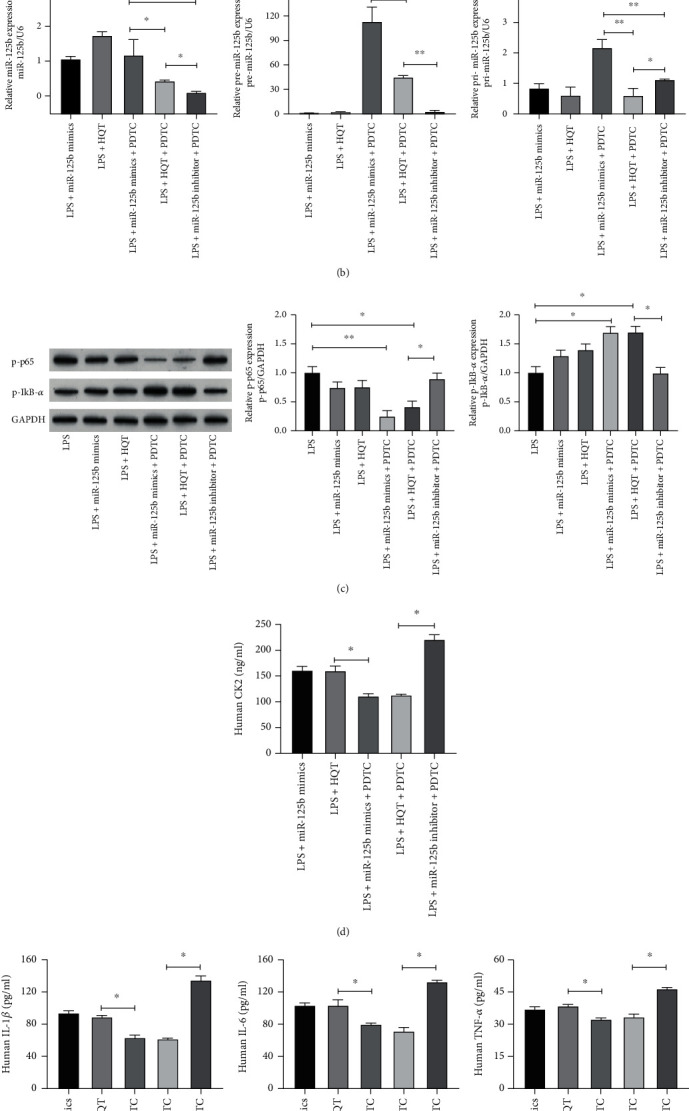
HQT ameliorates LPS-induced inflammation through upregulating miR-125b to suppress NF-*κ*B-induced inflammation by targeting CK2. (a) MH7A cells were treated with different concentrations of HQT such as 0, 0.1, 1, 10, and 100 mg/mL for 24 h; cell viability was evaluated via the CCK-8 assay. (b–e) MH7A cells were transfected with miR-125b mimics or miR-125b inhibitor and then treated with LPS and PDTC. (b) The mRNA expression of miR-125b, pre-miR-125b, and pri-miR-125b was evaluated via qRT-PCR. (c) The protein level of p-p65 and p-IkB-*α* was evaluated via western blot. (d) Human CK2 concentration in the cultured supernatant was measured by using an ELISA assay. (e) The cytokine protein levels including TNF-*α*, IL-1*β*, and IL-6 in the cellular supernatant were measured by using an ELISA assay. *N* = 3, ^∗^*p* < 0.05, ^∗∗^*p* < 0.01, and ^∗∗∗^*p* < 0.001.

To identify the best concentration of HQT-treating MH7A cells without cell toxicity *in vitro*, the cell viability of MH7A cells treated with different concentrations of HQT including 0, 0.1, 1, 10, and 100 mg/mL was evaluated via the CCK-8 assay. The results showed that only 0.1 mg/mL HQT had no effect on the cell viability of MH7A cells (Figure 5(a)). Thus, 0.1 mg/mL of HQT was utilized for the subsequent experiments without cell toxicity. Results of qPCR found that the levels of miR-125b, pre-miR-125b, and pri-miR-125b were evaluated via HQT in LPS-treated MH7A cells (*p* < 0.05) (Figure 5(b)), suggesting that HQT could promote the transcription of the host gene of miR-125b in MH7A cells. Interestingly, PDTC dramatically reduced HQT-increased levels of miR-125b, pre-miR-125b, and pri-miR-125b (Figure 5(b)), indicating that NF-*κ*B might contribute to the transcription of the host gene of miR-125b in MH7A cells under LPS condition.

Similar to miR-125b mimics, HQT treatment decreased the level of p-p65 and increased the p-IkB-*α* level in LPS-treated MH7A cells (*p* < 0.05), and PDTC enhanced the effect of miR-125b mimics and HQT on p-p65 and p-IkB-*α* (*p* < 0.05) (Figure 5(c)). By contrast, the miR-125b inhibitor reversed the inhibitory effect of HQT on p-p65 and p-IkB-*α* (*p* < 0.05) (Figure 5(c)). Besides, miR-125b mimics and HQT treatment significantly downregulated the CK2 expression in LPS-treated MH7A cells (*p* < 0.05), and PDTC enhanced the effect of miR-125b mimics and HQT on CK2 expression, whereas the miR-125b inhibitor reversed the effect of HQT on CK2 expression in MH7A cells (*p* < 0.05) (Figure 5(d)).

Consistent with the effect of miR-125b mimics on inflammatory cytokine production, HQT treatment reduced the production of TNF-*α*, IL-1*β*, and IL-6 in LPS-treated MH7A cells, and PDTC treatment further improved the inhibitory effect of miR-125b mimics and HQT on the production of TNF-*α*, IL-1*β*, and IL-6 (*p* < 0.05) (Figure 5(e)). By contrast, the miR-125b inhibitor abolished the inhibitory effect of HQT on the production of TNF-*α*, IL-1*β*, and IL-6 (*p* < 0.05) (Figure 5(e)). All these data together suggested that HQT could attenuate LPS-induced inflammation through upregulating miR-125b to suppress NF-*κ*B-induced inflammation by targeting CK2.

## 4. Discussion

This study investigated the mechanism of HQT in attenuating RA. Our results demonstrated that HQT could efficiently inhibit RA-induced inflammation by reducing the production of cytokines such as TNF-*α*, IL-1*β*, and IL-6. Moreover, HQT significantly upregulated the expression of miR-125b. Besides, CK2 was a target of miR-125b in MH7A cells. Furthermore, miR-125b inhibited LPS-induced NF-*κ*B activation, which is a downstream target of CK2. In addition, the NF-*κ*B inhibitor PDTC and IkB-*α* enhanced the inhibitory effect of miR-125b on the production of TNF-*α*, IL-1*β*, and IL-6.

HQT is a TCM herbal formula for treating RA developed by the Second Affiliated Hospital of Guangzhou University of Chinese Medicine [9], which is composed of the following natural materials: Salvia miltiorrhiza (Danshen), Dioscorea nipponica Makino (Chuanshanlong), Astragalus membranaceus (Huangqi), Paeonia lactiflora Pall (Baishao), Saussurea involucrata (Tianshanxuelian), Eucommia ulmoides Oliver (Duzhong), Rhizoma Drynariae (Gusuibu), Radix Dipsaci Asperoidis (Chuanxuduan), Radix Rehmanniae Preparata (Shudi), and Glycyrrhizae Radix et Rhizoma (Gancao) (Table 1). Our results demonstrated that HQT upregulated the miR-125b expression in RA. However, the underlying mechanism was not investigated in this study.

Salvia miltiorrhiza (Danshen) is one of the most widely used TCM. Previous studies have revealed that Salvia miltiorrhiza (Danshen) or the constituents of Salvia miltiorrhiza attenuate RA through inhibiting inflammatory response. For example, Tanshinone IIA (Tan IIA), one of the major phytochemicals extracted from Salvia miltiorrhiza (Danshen), suppresses cell proliferation and inflammatory cytokine production in FLS to ameliorate RA-induced inflammatory response [31]. Salvianolic acid B (SB) is a main active phytoconstituent of Salvia miltiorrhiza (Danshen), which reduces levels of the inflammatory mediator through downregulating NF-*κ*B in a RA rat model [32]. Numerous studies have indicated that Salvia miltiorrhiza-derived miRNAs contribute to cellular progress in mammalian cells. For instance, Salvia miltiorrhiza-derived sal-miR-58 suppresses chronic angiotensin II- (Ang II-) induced inflammatory response in mouse vascular smooth muscle cells (VSMCs) via triggering autophagy [33]. Besides, Salvia miltiorrhiza-derived sal-miR-1 and 3 inhibit vascular remodeling through suppressing the migration of VSMC by increasing the Krüppel-like factor 4 (KLF4) protein level via targeting OTU deubiquitinase 7B (OTUD7B) [34]. Therefore, these studies suggest that HQT components (such as Salvia miltiorrhiza-derived miRNA which is conserved to hsa-miR-125b) may enter FLS to increase the hsa-miR-125b level.

Besides, numerous components of HQT could regulate the expression of endogenous miRNAs in mammalian cells. It has been reported that Tan IIA downregulates mmu-miR-375 to increase the KLF4 protein expression and ameliorate atherosclerosis in ApoE knockout mice [34]. Moreover, Tan IIA suppresses VSMC inflammation and proliferation by decreasing the miR-712-5p level [35]. Furthermore, Tanshinone inhibits lung cancer cell proliferation through upregulating let-7a-5p [36]. Except for Salvia miltiorrhiza (Danshen) or the components of Salvia miltiorrhiza (Danshen), dioscin extracted from Dioscorea nipponica Makino (Chuanshanlong) significantly increases the miR-125a-5p level in insulin-induced HepG2 cells to reduce gluconeogenesis and lipogenesis [37]. In addition, astragaloside IV derived from Astragalus membranaceus (Huangqi) elevates the miR-135a expression in cardiomyocytes to prevent cardiac fibrosis in rats [38]. All these studies suggest that components of HQT might directly regulate endogenous miRNAs of FLS.

Some studies have revealed that Salvia miltiorrhiza (Danshen) or the components of Salvia miltiorrhiza (Danshen) could modify the expression of transcription factors in mammalian cells. For example, Salvia miltiorrhiza (Danshen) enhances the expression of forkhead box P3 (Foxp3) in murine colitis [39]. Besides, sodium Tanshinone IIA sulfonate, an extract of Salvia miltiorrhiza (Danshen) increases the nuclear expression of nuclear factor, erythroid 2-like 2 (NFE2L2) to attenuate pulmonary fibrosis [40]. Thus, we guessed that HQT may enhance the transcription of the host gene of hsa-miR-125b by regulating the expression of transcription factors.

To date, the relationship between miR-125b and CK2 is not clear. Thus, this study revealed that CK2 is a target of miR-125b in RA for the first time. In fact, the knowledge of the relationship between miRNA and CK2 is limited. Only several studies have indicated the regulatory effect of miRNAs on the CK2 expression. For instance, miR-217 reduces ERK activation through targeting CK2 in ganglioglioma [41]. Besides, miR-186, miR-216b, miR-337-3p, and miR-760 together induce cellular senescence via targeting CK2 in colorectal cancer cells [42]. Similarly, miR-760 and miR-186 cooperatively trigger replicative senescence by targeting CK2 in lung fibroblast cells [43]. Therefore, our study could expand the knowledge of the regulatory effect of miRNAs on CK2 expression.

This study also found that miR-125b inhibited the production of TNF-*α*, IL-1*β*, and IL-6 through targeting CK2 in RA for the first time. Though the role of CK2 in inflammatory cytokine production in RA, the positive effect of CK2 on the production of TNF-*α*, IL-1*β*, and IL-6 have been revealed. It has been reported that Esculentoside A suppresses the production of TNF-*α* by CK2 [44]. Besides, inhibition of CK2 abrogates IL-1*β* production in monocytes [45]. In breast cancer, CK2 enhances IL-6 production to promote tumor progression [24]. Hence, the finding of this study that miR-125b inhibited the production of TNF-*α*, IL-1*β*, and IL-6 through targeting CK2 in RA is reliable.

## 5. Conclusion

In summary, our study revealed that HQT could attenuate RA through upregulating miR-125b to suppress NF-*κ*B-induced inflammation by targeting CK2. The findings of this study should facilitate investigating the mechanism of HQT on RA and discovering novel therapeutic targets for RA.

## Figures and Tables

**Table 1:** Main components of HQT.

Pinyin name	Latin name	Doses
Danshen	Salvia miltiorrhiza	20 g
Chuanshanlong	Dioscorea nipponica Makino	30 g
Huangqi	Astragalus membranaceus	30 g
Baishao	Paeonia lactiflora Pall	20 g
Tianshanxuelian	Saussurea involucrata	3 g
Duzhong	Eucommia ulmoides Oliver	20 g
Gusuibu	Rhizoma Drynariae	20 g
Chuanxuduan	Radix Dipsaci Asperoidis	15 g
Shudi	Radix Rehmanniae Preparata	15 g
Gancao	Glycyrrhizae Radix et Rhizoma	10 g

## Data Availability

The [data type] data used to support the findings of this study are included within the article.
